# Alphavirus Entry and Membrane Fusion

**DOI:** 10.3390/v2040796

**Published:** 2010-03-26

**Authors:** Margaret Kielian, Chantal Chanel-Vos, Maofu Liao

**Affiliations:** Department of Cell Biology, Albert Einstein College of Medicine, 1300 Morris Park Ave., Bronx, NY 10461, USA; E-Mails: chc2019@med.cornell.edu (C.C.-V.); maofu.liao@ucsf.edu (M.L.)

**Keywords:** virus entry, membrane fusion, alphavirus, class II fusion protein, endocytosis

## Abstract

The study of enveloped animal viruses has greatly advanced our understanding of the general properties of membrane fusion and of the specific pathways that viruses use to infect the host cell. The membrane fusion proteins of the alphaviruses and flaviviruses have many similarities in structure and function. As reviewed here, alphaviruses use receptor-mediated endocytic uptake and low pH-triggered membrane fusion to deliver their RNA genomes into the cytoplasm. Recent advances in understanding the biochemistry and structure of the alphavirus membrane fusion protein provide a clearer picture of this fusion reaction, including the protein’s conformational changes during fusion and the identification of key domains. These insights into the alphavirus fusion mechanism suggest new areas for experimental investigation and potential inhibitor strategies for anti-viral therapy.

## 1. Introduction

A key step in virus infection is the entry of the virus into the host cell, releasing the virus genome into the cytoplasm. The entry process involves initial interactions of the virus with attachment factors and/or specific receptors, which play an important role in species and tissue tropism. For enveloped viruses, the next step in entry involves the fusion of the virus membrane with a membrane of the host cell, mediated by specific membrane fusion protein(s) on the virus surface. Virus membrane fusion occurs either at the plasma membrane or within organelles of the endocytic pathway following uptake by the cell. The site of fusion is dictated primarily by the triggering mechanism of the virus fusion protein. Such triggering mechanisms include interactions with virus receptors and/or co-receptors, exposure to low pH in the endocytic pathway, and the combination of receptor interaction and low pH. There has been considerable interest in the various steps in virus entry both as targets for antiviral therapy and as experimental paradigms to study cellular membrane processes including endocytosis, membrane traffic, and membrane fusion [for review see 1,2–5].

The alphaviruses are a genus of the Togaviridae family containing about 26 virus species including the well-characterized Semliki Forest virus (SFV) and Sindbis virus (SIN) [for review see 6,7]. Alphaviruses have been important experimental tools in furthering our understanding of membrane protein biosynthesis and transport, endocytosis, and membrane fusion. This review will focus on the entry and membrane fusion reaction of the alphaviruses, particularly SFV. Previous reviews of the alphavirus entry pathway will be referenced to summarize aspects that are not covered here in depth due to space constraints.

## 2. Alphavirus architecture and the structure of the fusion machinery

Alphaviruses are small spherical viruses with an internal nucleocapsid containing the viral positive-sense RNA genome [7]. This core is enveloped by a lipid bilayer membrane containing 240 copies each of two glycoproteins, the E1 membrane fusion protein and the E2 protein. Both E1 and E2 are Type I transmembrane proteins of about 50 kDa, arranged in a T = 4 icosahedral lattice on the virus surface [8–11]. As discussed in more detail below, although E1 is the fusion protein, the dimeric interaction of E2 with E1 is important in regulating fusion as well as in the folding and transport of newly synthesized E1 to the plasma membrane where virus budding takes place.

**Figure 1.**
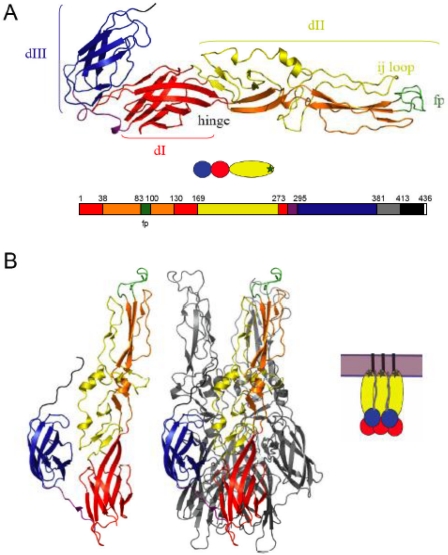
**The alphavirus membrane fusion protein E1 in the pre- and post-fusion conformations. (a)** The prefusion structure of the SFV E1* ectodomain. The three domains of E1 are shown, with DI in red, the two insertions (into DI) that comprise DII shown in yellow and orange, and DIII in blue. The fusion peptide loop (fp) at the tip of domain II is in green, the DI-DIII linker is in purple, and the positions of the ij loop and hinge are indicated. Below the structure is a cartoon view of E1* with DI, II and III colored in red, yellow and blue, respectively and the fusion loop shown as a green star. The bottom part of the panel shows a linear diagram of the E1 sequence colored to match the structure above and labeled to indicate the domain boundaries. The stem is shown in grey, the TM domain in black, and the cytoplasmic tail of E1 in white. [PDB 2ALA, references 10,12] **(b)** The post-fusion structure of the E1 ectodomain. One E1* subunit from the homotrimer is shown in the left panel, colored as in A. The E1* homotrimer is shown in the middle panel with one E1* subunit colored as in A and the other two E1* subunits shown in light grey. DIII (blue) and the stem (dark grey) extend along the core trimer towards the fusion loops (green). The cartoon in the right panel illustrates the hairpin conformation and depicts the full-length membrane-inserted E1 homotrimer with the complete stem (grey), TM domain (black) and fused membrane (light purple). [PDB 1RER, reference 13] Figure reprinted from Virology, 344, Kielian, M., Class II virus membrane fusion proteins, p. 40, Copyright (2006), with permission from Elsevier.

The prefusion structure of the proteolytically truncated ectodomain of SFV E1 (referred to here as E1*) has been determined (Figure 1A) [10,12]. E1 is an elongated molecule containing three domains (DI-DIII) composed almost exclusively of ß-strands. DI is the central domain, and contains two long insertions that form DII: insertion 1 from residues 38–130 and insertion 2 from residues 169–273. At the tip of each insertion is a loop connecting 2 ß-strands: the cd loop containing the hydrophobic fusion peptide loop (between ß-strands c and d), and the spatially adjacent ij loop (between ß-strands i and j). DIII has an immunoglobulin-like fold, and is connected to DI via a linker region. The C-terminus of DIII connects with the stem region and the transmembrane domain that anchors the ectodomain in the virus membrane. In the virus particle E1 lies tangential to the virus membrane and forms the icosahedral scaffold, while E2 is found in spike-like projecting domains that cover much of the E1 protein including the DII tip with the fusion loop [10–12].

These studies along with determination of the structures of the fusion proteins from the flaviviruses Tick-Borne Encephalitis (TBE) virus, dengue virus (DV), and West Nile virus (WNV) [14–19] revealed the important structural similarity of the fusion proteins of the alphaviruses and flaviviruses, which are frequently referred to as class II fusion proteins. In addition to their shared structural features, the alpha and flavivirus fusion proteins have a number of functional properties in common, including proteolytic processing of the companion subunit to activate fusion, an internal fusion peptide that forms a loop at the tip of the fusion protein, and a critical conversion from a dimer to a homotrimer (HT) during fusion [20]. The post-fusion conformation of all of the virus fusion proteins whose structure has been determined is a trimeric “hairpin” structure with the fusion peptide and transmembrane domain at the same end of the trimer [reviewed in 4,5,20,21]. The postfusion conformation and refolding reaction of the alphavirus E1 fusion protein will be discussed below.

## 3. The alphavirus entry pathway

### 3.1. Receptors and attachment factors

Alphaviruses are able to infect a wide range of species including mammals, birds, and insects, and are transmitted in nature by mosquito vectors [reviewed in 6,22]. The wide host range of alphaviruses may be due in part to the ability of these viruses to bind to different receptors on cells of different tissues and species. Early studies of alphavirus interaction with host cells revealed that virus binds to the plasma membrane of a number of cell types in the cold [reviewed in 23]. Binding of virus is saturable and is primarily mediated by the viral E2 protein. Bound virus can be removed by protease digestion under conditions that do not affect the virus but presumably digest the virus receptor. The protease required to remove bound virus varies depending on the cell type, again in keeping with the protease acting by digestion of cell surface proteins.

A number of cell surface molecules have been suggested to act as alphavirus receptors and/or attachment factors, including the high-affinity laminin receptor, the class I major histocompatibility antigen, α1ß1 integrin [24], several proteins identified by monoclonal antibodies, cell surface heparan sulfate (HS), and DC-SIGN and L-SIGN [reviewed in references 6,22,25,26]. HS binding is clearly mediated by the E2 protein and is increased in tissue culture-adapted alphaviruses [26–30]. However, viruses that have adapted to efficiently bind HS are attenuated when tested *in vivo* [31,32], and thus the *in vivo* role of HS-binding is unclear [33]. Binding to HS also occurs with high efficiency in viruses containing unprocessed p62, the precursor to E2, since the intact cleavage site can interact with HS [34–36].

DC-SIGN/L-SIGN are C-type lectins that bind to mannose-rich carbohydrate structures [37]. Interestingly, mosquito cells synthesize high mannose and Man_3_GlcNAc_2_ structures [38], and virus produced from these cells shows strongly increased binding and infection on cells expressing DC-SIGN/L-SIGN [25]. Thus transmission of alphaviruses from infected mosquitoes to vertebrate hosts may be enhanced by the interaction of mannose-rich carbohydrate on the mosquito-derived virus with DC-SIGN/L-SIGN molecules on dendritic cells and other cell types [see 25 for discussion]. The role of DC-SIGN/L-SIGN on expressing cells in mediating trans-infection of cells not expressing these lectins is not yet clear, and could also play an important role during *in vivo* infection.

### 3.2. Receptor-mediated endocytosis of alphaviruses

Studies with SFV first demonstrated the pathway of endocytic uptake followed by low pH-triggered fusion [39], and the general features of this pathway are now known to be used by a number of enveloped animal viruses [40]. The evidence for alphavirus infection via this pathway will be summarized here and in Table 1. As there is an extensive literature on this topic, the reader is also referred to a number of reviews [6,22,23,41,42].

Following binding to the plasma membrane through the receptors discussed above, alphaviruses such as SFV are internalized via the clathrin-mediated endocytic pathway. This pathway is used by cells to take up many physiologically important ligands such as low density lipoproteins, transferrin, growth factors, hormones, *etc.* [66]. Quantitative experiments showed that SFV does not appear to induce or increase the formation of clathrin-coated vesicles [43], and thus virus entry is believed to occur by piggy-backing on a receptor molecule that is being constitutively internalized. The initial endocytic uptake of SFV can be blocked by microinjection of anti-clathrin antibodies [67], or by dominant-negative versions of key proteins of the endocytic pathway such as dynamin and eps15 [inhibitors of the endocytic pathway are reviewed in 42]. Both of these dominant-negative proteins act to inhibit alphavirus infection while permitting infection by other viruses that infect via alternative routes [45–47].

Uptake of SFV is very rapid at 37 °C, with a half-time of 3–10 min depending on the cell type [51,68]. Once endocytosed, the clathrin coat on the virus-containing vesicles is rapidly removed and the virus is delivered to the early endosome compartment. Endocytic uptake is blocked by keeping cells on ice, but uptake, delivery to the early endosome, and virus fusion and infection all occur in cells incubated at 20°C [69]. By removing extracellular SFV with protease digestion, it was shown that endocytosed virus infects from within the endosome compartment [44]. Recent studies also demonstrated that blocking the formation of early endosomes by expression of dominant-negative rab5 specifically inhibited alphavirus infection in mammalian and mosquito cells [48,70].

### 3.3. Low pH-triggered fusion in endosomes

A critical feature of the endosome compartment is the presence of an ATP-dependent proton pump in the endosome membrane. This protein complex, termed the vacuolar ATPase, acts to acidify the lumen of endosomes and lysosomes, and is important in receptor-ligand trafficking [71]. As ligands including viruses transit through the endocytic pathway from early to late endosomes and then to lysosomes, they are exposed to increasingly low pH, ranging from a pH of ∼6.0–6.5 in the early endosome, late endosome pH of ∼5.0–6.0 and then to a lysosomal pH range of ∼4.6–5.0 [68,71]. For commonly used wild type SFV strains, fusion occurs within the early endosome compartment with a fusion threshold of ∼pH 6.2 [51]. However, alphaviruses can differ markedly in the pH required to trigger fusion, with for example the Toto 1101 strain of SIN having a fusion threshold of ∼pH 5.6 [49]. The pH required to trigger fusion can also be modulated by specific mutations in the virus envelope proteins (see section 5 below). The organellar location of the virus fusion reaction thus depends on the pH threshold required to trigger fusion, and may be important for viral fitness. Interestingly, evidence suggests that while many cells can be infected by low pH-triggered fusion of virus bound to the plasma membrane [e.g., 65,86], in some cell types plasma membrane fusion does not result in infection although the cells can be infected by the normal endocytic route [72]. Thus, at least in some cell types, endocytic uptake may be important to bring the virus to an intracellular site where productive replication can occur. The intracellular location of the fusion reaction also explains the absence of alphavirus proteins in the cell plasma membrane immediately after infection, in contrast to a virus such as Sendai that fuses directly with the plasma membrane [73].

The role of endosomal low pH in triggering alphavirus fusion has been studied extensively by using various inhibitors of endosomal acidification, as summarized in Table 1 and references therein. Three different classes of acidification inhibitors have been shown to block SFV infection: weak bases, proton ionophores, and specific inhibitors of the vacuolar ATPase. For such inhibitor studies, it is important to rule out potential secondary effects and to monitor infection using sensitive linear assays, ideally of early steps in the infection pathway. All three inhibitor classes have been shown to block infection specifically when present during virus endocytic uptake but not during receptor binding or early replication steps. Biochemical studies demonstrate that all three classes of inhibitors act to block low pH-dependent conformational changes in the alphavirus fusion protein (see also section 4 below) and the release of the nucleocapsid into the cytoplasm. Experiments also demonstrate that the vacuolar ATPase inhibitors specifically block alphavirus-membrane fusion as detected by following a fluorescent lipid probe in the viral membrane [46,52]. In agreement with these results, infection of retroviral cores pseudotyped with alphavirus envelope proteins is blocked by several acidification inhibitors while infection of the same cores pseudotyped with envelope proteins from viruses that are not low pH-triggered is unaffected [53,70].

## 4. Alphavirus membrane fusion

### 4.1. General properties of alphavirus-membrane fusion

The general features of alphavirus fusion observed during virus entry have also been observed using numerous *in vitro* methods including assays of virus-cell fusion, lipid mixing and content mixing assays for virus-liposome fusion, and assays of fusion of virus-infected or E1-expressing cells with target cells or with a planar lipid bilayer (Table I). Mutants that have a shifted pH threshold *in vitro* display altered entry kinetics and differential sensitivity to acidification inhibitors *in vivo* [e.g., 74], while mutants that are blocked for fusion *in vitro* are blocked in fusion during entry and are non-infectious [e.g., 62,63]. Key features of alphavirus fusion *in vitro* and *in vivo* are the involvement of a low pH trigger and the promotion of fusion by sterol. Fusion with protein-free lipid bilayers occurs very rapidly and efficiently at low pH, resulting in fusion of most of the input virus within seconds at 37 °C [e.g., 58]. Virus-receptor binding thus does not appear to be critically required for fusion but may play a role during virus entry *in vivo*, where it may be involved in helping to trigger fusion and E1 conformational changes [75,76].

### 4.2. Lipid dependence

Alphavirus fusion is promoted by the presence of cholesterol and sphingolipid in the target membrane, and the role of these two lipids will be reviewed below [see also 77,78].

Role of cholesterol. A role for target membrane cholesterol in alphavirus fusion was first demonstrated using *in vitro* assays of low pH-triggered virus-liposome association [79] and content and lipid mixing assays of virus-liposome fusion [58,60,80]. Studies with cholesterol analogs demonstrated that the sterol 3ß-hydroxyl group is critical while other aspects of sterol structure are not, suggesting that cholesterol does not act via bulk effects on physical properties of the membrane such as membrane fluidity [80]. Fusion was optimal at ratios of ∼1 cholesterol per 2 phospholipid molecules [60], levels that are similar to those found in eukaryotic plasma membranes [81].

The role of cholesterol *in vivo* was addressed using mosquito cells, which like all insect cells are cholesterol auxotrophs [82], and can be cultured under highly cholesterol-depleted conditions without deleterious effects or compensatory changes in lipid composition [83]. Primary SFV and Sindbis infection of cholesterol-depleted mosquito cells is reduced by ∼3–4 logs compared to non-depleted cells, and infection by low pH-triggered fusion with the cholesterol-depleted cell membrane is decreased by ∼4 logs [84–87]. In contrast, virus-receptor binding, endocytic uptake and endosome acidification are unaltered in depleted cells. In addition, infection by direct transfection of SFV RNA or infection by the cholesterol-independent rhabdovirus vesicular stomatitis virus is unimpaired. Efficient alphavirus infection is restored by repletion of depleted cells with cholesterol but not cholestenone, suggesting that the requirement for the 3ß-hydroxyl group is similar in liposomes and cell membranes [84]. The relatively rapid reversal of inhibition by repletion with purified cholesterol demonstrates that this component alone is responsible for the effect. This is in keeping with the lack of cholesterol biosynthesis by insect cells, and their ability to synthesize other membrane lipids such as phospholipids [82,83]. Alphavirus budding is also specifically inhibited in cholesterol-depleted cells [85,87,88]. The decrease in budding was observed with biochemical assays of virus particle assembly. The role, if any, that cholesterol in the virus membrane might play in infectivity is unclear, although several studies have documented that virus particles produced in sterol-depleted cells are less stable to physical stress such as gradient sedimentation [86,89].

Mosquitoes, the vector for alphaviruses in the wild, are cholesterol auxotrophs that require dietary sterol to develop into adults [82]. Alphavirus infection of mosquitoes can occur via a blood meal from an infected animal, or by vertical transmission or intrathoracic injection in the absence of a blood meal [90,91]. Under the latter two conditions, the residual cholesterol content of the relevant tissues may be high enough to permit virus fusion and infection. While insects are cholesterol auxotrophs, they can metabolize plant sterols known as phytosterols to cholesterol and thus there are several avenues that can result in 3ß-hydroxysterol in insect cell membranes. It is also possible that *in vivo* the lipid composition of mosquito membranes could support fusion even under cholesterol-poor conditions. Under some extended conditions of cholesterol-depletion, mosquito cells in tissue culture were found to become relatively more permissive for SFV infection, fusion, and budding, even though cholesterol levels remained very low [92]. While the mechanism of this alteration is unclear, it suggests that modifications in membrane lipid composition could partially compensate for the effect of cholesterol depletion on SFV fusion.

Role of sphingolipid. Fusion of SFV or SIN virus or cells expressing SFV envelope proteins is promoted by the presence of ∼2 mole % sphingolipid in the target lipid bilayer [61,93,94]. Ceramide is the minimal sphingolipid that supports fusion [93]. The lack of cells deficient in ceramide and sphingolipid has prevented studies of virus infection. Although both sphingolipids and cholesterol are involved in the formation of detergent-resistant membrane microdomains often referred to as rafts [95], the ability of these lipids to support fusion in lipid-mixing assays does not correlate with their efficiency in generating detergent-resistant membrane domains [96]. The role of sphingolipids in alphavirus fusion could be through effects on the accessibility of cholesterol in the target membrane.

### 4.3. Conformational changes during fusion

**Figure 2.**
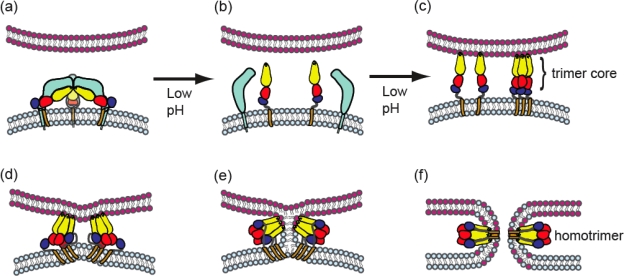
**Model for stages of the alphavirus membrane fusion reaction. (a)** Virus particle in the pre-fusion state. The virus membrane, depicted in light blue, contains a trimer of E2-E1 heterodimers, with E2 in light blue and E1 colored as in Figure 1. The target membrane is shown in pink. The fusion protein E1 is in a metastable conformation. **(b)** Triggering. Upon exposure to low pH, dissociation of the E2-E1 heterodimer occurs, exposing the E1 fusion loop. The disposition of E2 after heterodimer dissociation is unknown. **(c)** The fusion loop inserts in the target membrane through a low pH and cholesterol-dependent mechanism. A core trimer is formed by DI and DII. **(d-e)**. In a pH-independent interaction, DIII and the stem region are folded against the core trimer in the groove formed by two E1 proteins. The distortion of the target membrane by fusion loop insertion, the fold-back of DIII and stem, and the cooperative action of several trimers (of which only two are shown) are proposed to provide the force to mediate membrane fusion. **(e)** Fusion proceeds through a hemifusion step in which the two outer leaflets merge. **(f)** E1 forms the final stable post-fusion homotrimer, in which the fusion loops and the transmembrane domains are located at the same side of the molecule. Concomitantly, this refolding drives complete fusion via formation of the fusion pore. Figure reprinted from Trends in Microbiology, 17, Sanchez-San Martin, C., Liu, C. Y., and Kielian, M., Dealing with low pH: entry and exit of alphaviruses and flaviviruses, p. 517, Copyright (2009), with permission from Elsevier.

The overall alphavirus membrane fusion pathway involves dissociation of E1 from E2, E1 insertion into the target membrane, and formation of a stable E1 homotrimer (Figure1B, Figure 2). The general features of this pathway will be summarized here.

E2/E1 dimer dissociation. Binding of virus to the surface of cells followed by warming of the complex to 37 °C results in the exposure of previously hidden monoclonal antibody (mAb) binding sites termed “transitional epitopes” [76,97,98]. These epitopes are found on both E1 and E2, and their exposure may represent an early conformational change due to receptor binding or target membrane interaction.

Upon exposure to endosomal low pH or *in vitro* low pH treatment, the dimeric interaction of E1 and E2 is destabilized, as detected by the dissociation of the normally stable dimer upon solubilization in non-ionic detergent [99,100]. Dimer dissociation is an important regulator of the E1 fusion protein, as discussed in more detail in section 5.1. Dissociation is a relatively early event in fusion that precedes E1-membrane insertion and fusogenic conformational changes, as shown by its triggering at a higher pH threshold [99] with faster kinetics [101]. A mAb that maps to the fusion peptide was used to demonstrate that the fusion loop becomes exposed under conditions of dimer dissociation [102,103] (see also section 5.2 below).

E1-membrane insertion. Following dimer dissociation, virus associates with the target membrane, resulting in co-migration with target liposomes on sucrose floatation gradients. This interaction is due to the initial insertion of the fusion loop into the membrane (usually followed by membrane fusion), and is promoted by low pH and cholesterol [80].

Homotrimer formation. A key step in alphavirus membrane fusion is the refolding of E1 to the postfusion homotrimer (HT), a conformation that can be detected by assays including chemical crosslinking, sucrose gradient sedimentation, and the relative resistance of the HT to trypsin digestion and to dissociation by SDS-sample buffer at 30°C. The evidence for the role of the HT in alphavirus fusion is summarized in Table 2. The E1 HT was first observed during virus uptake into cells as the low pH-dependent formation of a trypsin-resistant E1 oligomer [100]. Subsequent analysis showed that the oligomer is an E1 homotrimer [104]. In retrospect, the low pH-induced trypsin-resistant form of E1 that had been previously characterized *in vivo* and *in vitro* thus reflects the presence of the E1 HT [51,105]. A mutation in the SFV fusion peptide, G91D, blocks complete fusion and hemi-fusion (mixing of the outer leaflets of the viral and target membranes), virus infection, and trimer formation [62,106], indicating the critical involvement of the homotrimer in fusion. Extensive studies also showed that virus fusion and the formation of the E1 HT have similar kinetics and pH dependence under a variety of conditions [35,74,107].

The kinetics of virus-membrane association are very close to those of E1 trimerization, and it was originally suggested that trimerization occurred prior to E1-membrane insertion [58]. However, studies with Zn^2+^, an inhibitor of fusion [108], and with several SFV E1 mutants [62,64], demonstrated that virus-liposome binding can occur under conditions in which formation of the final postfusion E1 homotrimer is inefficient or blocked. The stages of HT formation are presented in detail below.

Acid-epitope exposure. Low pH-treatment also induces the exposure of acid conformation-specific monoclonal antibody (mAb) epitopes on E1. Although various epitopes on E1 are hidden in the assembled virus particle (by interactions with E2, for example), the epitopes specific to the acid conformation are not exposed by detergent solubilization of the virus particle or by E1 isolation, but require pretreatment of the virus at a pH similar to that which triggers fusion [104,109]. All four antibodies of this type cross-compete for binding to E1, suggesting similar or spatially-related binding sites [110]. One of the antibodies was mapped by selecting an antibody-resistant mutant, which was shown to have a substitution of E1 G157R [110]. This mutation in DI decreases the binding of all four acid-specific mAbs, confirming their related binding sites. Exposure of this region of E1 was also observed in mapping studies of low pH-treated whole virus particles [111]. E1 trimerization and acid-epitope exposure occur with comparable kinetics [58], and biochemical analysis showed that the E1 homotrimer reacts with the acid-specific mAbs [112]. However, the acid-specific epitope can also become exposed upon low pH treatment of the trimerization-negative G91D mutant, suggesting that epitope exposure is distinct from formation of the final postfusion HT [62].

### 4.4. E1 ectodomain studies

The E1 ectodomain is a soluble form of E1 that is missing the TM domain and about two-thirds of the stem region that connects the TM domain to DIII. E1 ectodomains have been prepared by protease cleavage of virus E1 to give forms termed either E1* [105] or E1ΔS [113]. A similar soluble form, E1s, is produced by virus-infected cells, particularly under conditions in which budding is inefficient [114,115]. A drosophila cell expression system has recently been used to produce E1’, which is truncated at the end of DIII [116]. The ectodomains are monomeric [74,105,112,116], and thus dimer dissociation is not required for the response to low pH. All of the E1 ectodomains undergo low pH-dependent conformational changes similar to those observed for viral E1, including insertion into target liposomes, formation of a trypsin- and SDS-resistant homotrimer, and exposure of acid-specific epitopes [105,109,112,116]. These conformational changes are strongly promoted by the presence of cholesterol and sphingolipid in the target liposome, and the target membrane interaction helps to orient and concentrate the monomeric E1 ectodomains to promote trimerization. The E1*HT was used for structural characterization of the HT, as summarized below.

### 4.5. E1 homotrimer structure

Electron microscopy studies revealed that the membrane-inserted E1* homotrimers project perpendicularly from the target membrane, and that E1*HT forms clusters on liposomes even at a high lipid-to-protein ratio [117]. The clusters form rings of 5–6 trimers and change the curvature of the target membrane. Thus, during insertion/trimerization E1 reorients significantly from its original tangential position on the virus particle and inserts into the membrane via a cooperative process. Such cooperative insertion and effects on membrane curvature would presumably play an important role in the SFV fusion reaction.

The membrane-inserted E1*HT was solubilized, purified, and the three-dimensional (3D) structure determined [13,118]. The E1* domains I, II and III essentially maintain their original folds in the HT conformation, but the interactions between domains change significantly and the molecule adopts a folded-back conformation (Figure 1B). DII rotates by ∼15° about a hinge region, resulting in a straight continuous rod comprised of domains I and II which interacts extensively to form the central core trimer. DIII and the stem region move ∼37 Å towards the fusion loop and interact with the HT core, resulting in a trimeric hairpin configuration in which the fusion loops and TM domains are at the same end of the trimer. The structures of flavivirus homotrimers show a similar central trimer of DI/II and an outer layer formed by the fold-back of DIII [119,120].

Observed interactions between fusion peptides in adjacent E1* trimers suggest that five or six trimers may interact during fusion [103]. The HT interactions visualized by microscopy and in the 3D structure are also in agreement with oligomers that were observed in gel electrophoresis [121], and with the studies of truncated E1 proteins described below [116].

### 4.6. Properties of the alphavirus fusion pore

The initial aqueous connection between the virus membrane and the target membrane is termed the fusion pore (Figure 2f). This connection has been studied using cells expressing the alphavirus glycoproteins and target cells or planar bilayer target membranes [56,57,61,122]. Similar to other virus fusion reactions, alphavirus membrane fusion proceeds through a hemifusion intermediate in which the outer leaflets of the virus and target membranes mix (Figure 2e). Subsequent formation of the initial fusion pore connection is rapid (on average within ∼10 sec of shift to low pH) and triggered specifically by low pH. The pore increases in size as fusion is completed. Transfer of a membrane dye to the target membrane is initiated shortly after pore opening. The planar bilayer studies revealed low pH-dependent conductance increases that occur even in the absence of cholesterol or in the presence of fusion inhibitors such as Zn^2+^ [61]. These “leaks” may represent reversible E1-membrane interactions.

Studies of cells expressing the alphavirus glycoproteins also indicate a role of *trans*-negative membrane potential in pore formation [61,122]. Optimal fusion occurs when the planar bilayer voltage is maintained at −40 mV, with the sign indicating negativity on the side of the planar membrane opposite to the cells [61]. Such a *trans*-negative membrane potential would be in keeping with the membrane potential expected across the endosome membrane, which has a high lumenal proton concentration compared to the cytoplasm. Trans-negative membrane potential is required for steps following the creation of the hemifusion intermediate [122]. It will be interesting to evaluate the role of *trans*-negative membrane potential in virus fusion and infection [123].

As in other virus systems, alphavirus fusion pores are relatively large aqueous channels connecting the fusing membranes. A number of studies suggest that even in the absence of fusion the E1 protein can cause a different type of ion-permeable pore [123–127]. Such ion-permeable pores are hypothesized to represent E1 insertion into the virus membrane or the membrane of the E1-expressing cell in the absence of a target membrane. However, this type of pore does not seem to play a critical role in alphavirus fusion and infection [127], perhaps in keeping with the observed non-leakiness of the alphavirus fusion reaction [61,128].

The alphavirus genome encodes a region termed “6K” between the p62 and E1 proteins [7]. From this region are derived the small hydrophobic peptide 6K [129] and the recently described TF peptide [130], which both appear to be incorporated into alphavirus particles at low levels and could cause permeability effects on the virus membrane. SFV with a 6K deletion is still fusogenic and infectious although the efficiency of lipid mixing is decreased [131,132]. Further studies are needed to address the potential functions of 6K and TF during virus entry.

## 5. Molecular analysis of the alphavirus membrane fusion reaction

### 5.1. Regulatory role of p62/E2

Cleavage activation of the heterodimer. E2 is synthesized as a precursor termed p62 or PE2, and associates as a heterodimer with E1within the ER [reviewed in 6,7]. This heterodimeric interaction is important for correct protein folding and virus budding [133]. The p62 precursor is processed late in the secretory pathway by cellular furin, which cleaves after a tetrabasic motif (RXR/KR) to produce E2 and a peripheral polypeptide E3 [36]. The E3 peptide remains associated with E2 in SFV but not, for example, in SIN, and thus E3 does not play a role in fusion. However, the processing of p62 is important in the regulation of the fusion activity of E1 as addressed by the study of viruses containing unprocessed p62.

A variety of mutations have been used to block furin cleavage and allow characterization of the resulting p62 virus. The furin site in SFV p62 has been mutated from RHRR to RHRL or to SHQL [107,134]. The furin site in Venezuelan equine encephalitis virus (VEE) has been deleted or mutated from RKRR to RKRD (or several other residues at the −1 position) [135]. The Sindbis virus mutant TRSB-N indirectly abrogates PE2 processing by the substitution of Asn for Arg at E2 position 1, which creates a signal for N-linked glycosylation [136,137]. In addition, infection of furin-deficient FD11 cells with wild type SFV produces p62 virus with the wt sequence [36]. p62 maturation into E2 and E3 is not required for virus assembly and release and all of these viruses bud efficiently. However, all have severely reduced infectivity due to decreased fusion activity. Both fusion activity and infection are recovered if p62 is cleaved by exogenous furin or trypsin digestion [36,107], or by endocytosis of p62 wt virus into cells that express furin [36]. The cleavage site mutations produce additional inhibitory effects on virus-cell binding [35,107].

The inhibition of p62-virus fusion activity is due to a strong acid shift in the pH of fusion to a threshold of pH 5.0 or below [35,36,107]. While the E2/E1 heterodimer is destabilized at the pH range of early endosomes, the p62/E1 heterodimer requires a pH of ∼ 5.0 or lower to trigger its dissociation [99]. The shifted pH threshold of p62/E1 dimer dissociation causes a shift in the pH threshold for the subsequent conformational changes in E1 such as homotrimer formation, thus affecting the pH of fusion [35,36,107]. The relatively acidic pH threshold of the p62/E1 dimer may be important in preventing the premature activation of E1 within the acidic environment of the secretory pathway. Furin cleavage late in the exocytic pathway would then prime the dimer for dissociation and fusion at endosomal pH. Cryo-electron microscopy reveals a dramatic but localized conformational change due to p62 cleavage in SFV [138], which may reflect this difference in dimer interaction before and after cleavage.

Mutations that affect heterodimer stability. A variety of approaches have been used to isolate mutations that affect heterodimer stability and that thus may identify sites of dimer interaction (Table 3). One method has been to select for second-site “resuscitating” mutations that allow growth and infectivity of viruses with the above-described mutations in the p62 cleavage site [35,135,137,139]. Alternatively, SFV mutants termed *pci* mutants (p62 cleavage-independent) have been isolated based on their increased growth in furin-deficient cells [140]. Both of these approaches yielded mutations that increase the infectivity of p62 virus and allow fusion at a pH threshold closer to that of E2 virus. While some of the mutations are not well-understood, several mutants were shown to have recovered the ability to form E1 homotrimers at mildly acidic pH [35,140,141]. This is presumably due to a decrease in the stability of the p62-E1 dimer, as has been demonstrated for mutants resulting from each approach [139–141]. The second-site mutations that compensate for defective p62 cleavage sites tend to be specific for the virus sequence used for the selection, perhaps suggesting that they are compensating for pleiotropic effects of the parental mutations on fusion and binding [135,137].

Sequence analysis demonstrated that most of the resuscitating mutations are located in the E2 sequence, primarily in the N-terminal half (Table 3). Thus, the dimer interactions most important for fusion regulation appear to lie in the N-terminal half of E2. Clearly more structural information on E2 and p62 is required to interpret the location of the mutations in the context of the virus and envelope protein structure. It will also be important to determine the effects of the mutations on the E2/E1 dimer, as an approach to understanding the changes that take place upon p62 processing and how they affect heterodimer stability and fusion regulation. Studies of one of the pci mutants demonstrated that the point mutation that destabilized the p62/E1 dimer indeed had a similar effect on the E2/E1 dimer, and shifted the pH threshold for fusion and E1 trimerization of E2 virus [141].

In a complementary approach, an SFV mutant that is resistant to fusion at mildly acid pH has been isolated and characterized [51,68,144]. This mutant, termed *fus*-1, exhibits a pH threshold for fusion of ∼pH 5.5 instead of the wt level of pH 6.2. This fusion phenotype is due to a single mutation in the E2 protein, threonine 12 to isoleucine [74]. The mutation acts by making the mature E2/E1 dimer more acid-stable, which in turn results in a more acidic pH requirement for the subsequent fusogenic conformational changes in E1 [74,141].

Chimeric viruses containing PE2 from SIN and 6K and E1 from Ross River virus have also been used to assess the importance of the heterodimer interaction in budding and fusion. The compensatory mutations obtained by this method are summarized in Table 3 and references therein.

### 5.2. E1 fusion loop and target membrane interaction

While the pH-dependent E1-E2 heterodimer interaction is critical in regulating the activity of E1, it is clear that regulation of fusion involves more than just dimer dissociation and the exposure of the E1 fusion loop. This additional regulation is reflected in the independent response of E1 to low pH. For example, studies of the *fus-1* pH-shift mutant demonstrated that the monomeric E1* ectodomain has a pH threshold considerably higher than that observed when it is associated with the acid-shifted mutant E2 protein [74]. Thus it is important to consider the mechanism of E1 membrane insertion and trimerization independent from their regulation by interaction with E2.

Insertion of E1 into the target membrane via the fusion peptide loop. The fusion peptide of alphavirus E1 was first identified by its hydrophobic and highly conserved nature as an internal region between residues K79 and D97 [145]. While the exact boundaries of the region that inserts into the target membrane are not known, the structure of E1 shows that the cd loop includes ∼residues 83–100 [10,12]. Using a monoclonal antibody that maps to E1 residues 85 to 95 (MAb E1f) as a probe, it is clear that the fusion loop is shielded by E2 on the native virus particle, becomes exposed after low pH treatment dissociates the E2/E1 dimer, and inserts into the target membrane [102,103]. However, exposure and membrane insertion of the fusion loop are not obligatorily coupled, as this region is fully exposed on monomeric E1 ectodomains [12,103], yet its stable membrane interaction requires incubation of E1 at low pH in the presence of target membranes containing cholesterol [103,116].

The membrane-inserted E1 ectodomain has interesting properties suggesting a possible association with cholesterol. E1 associates strongly with detergent-resistant membrane microdomains (DRM) that are enriched in cholesterol and sphingolipid [146], and E1 is extracted from membranes by treatment with the cholesterol acceptor methyl ß-cyclodextrin (MßCD). In contrast, membrane fusion of both influenza virus and DV is cholesterol-independent, and the membrane-inserted ectodomains of their fusion proteins do not associate with DRM and are resistant to MßCD extraction [78,146]. Recent studies demonstrated that the membrane-inserted SFV E1 ectodomain is specifically labeled with a photoactivatable form of cholesterol, while the DV E ectodomain does not label [78]. This result provides the first direct evidence for an E1-cholesterol interaction, and suggests a possible mechanism for the role of cholesterol in alphavirus-membrane fusion.

The function of specific residues in the fusion peptide loop. Mutagenesis of expressed SFV envelope proteins was used to test the role of the E1 fusion loop in cell-cell fusion [147]. The mutations fall into four categories according to their phenotypes: i. K79Q and M88L have no effect on fusion activity; ii. D75A, G83A and G91A acquire a lower pH threshold for fusion; iii. P86D, G91P and a deletion of residues 83 to 92 cause significant protein misfolding and block E1 transport out of the ER; iv. G91D completely blocks cell-cell fusion activity without affecting E1 biosynthesis and transport.

More detailed studies of G91 were performed using the SFV infectious clone to generate mutant viruses [62,106,148]. G91A virus shows limited secondary infection and an acid-shifted pH threshold for virus-cell fusion. G91D virus is non-infectious and is inactive in both content mixing and lipid mixing assays of membrane fusion. Both the G91A and G91D E1 proteins respond to low pH as measured by reactivity with an acid-conformation specific mAb at the same pH threshold as wt virus. The G91A virus also binds target liposomes and forms the E1 homotrimer, but both processes show an acid-shifted pH threshold and reduced efficiency. In contrast, G91D virus efficiently associates with liposomes, but is blocked in E1 homotrimer formation. Revertant analysis showed that G91 is essential for SFV viability [62,106]. Interestingly, although a glycine at position 83 is also highly conserved, a G83D mutation produces fully functional virus that shows normal fusion and E1 trimerization [106]. This suggests that G83 may lie outside the border of the critical region of the fusion loop.

### 5.3. Characterization of the E1 homotrimer

Biochemical analysis. The E1HT is considerably more stable than the prefusion conformation of E1 [149]. This is analogous to the differences in stability of the pre-fusion “class I” fusion proteins and their post-fusion conformations, which have a central α-helical coiled-coil domain. The fusion reactions of the class I viruses influenza virus and simian virus 5 (SV5) can be induced through destabilization of the prefusion form by treatment at high temperature or with denaturants such as urea [150–152]. However, similar destabilizing conditions do not induce SFV E1 trimerization or even initial lipid mixing [149], [see also 153 for similar results with a flavivirus fusion protein]. These results suggest potential differences in the energy barrier or lipid requirements for the fusogenic conformational changes of these two groups of viral fusion proteins.

The biochemical properties of the E1HT and E1*HT have been extensively characterized and compared [121,149]. Both the full-length and ectodomain homotrimer are very stable and are relatively resistant to dissociation by heat, urea or SDS at 30°C, and to protease digestion even after treatment with 5 M urea. Treatment with the reducing agent ß-mercaptoethanol selectively disrupts the HT structure, allowing proteolytic digestion within the fusion loop region and the ij loop (Figure 1) and releasing the bulk of the protein from the target membrane as a water-soluble trimer [121].

Residues involved in E1 pH dependence. While the dissociation of the E2-E1 dimer interaction is a key initial response to low pH, the fusion reaction carried out by E1 is also promoted by low pH [reviewed in reference 21]. The pH-dependence of E1 is probably complex and may involve intial triggering and subsequent E1 refolding during trimerization. A role of conserved E1 histidine residues has been suggested [10,154], and was tested by mutagenesis of the SFV infectious clone [155]. The H125A, H331A and H331A/H333A virus mutants have wild type growth properties and show no or modest changes in pH dependence. However, alanine substitution of the conserved H3 residue in DI impairs virus growth and decreases the efficiency and the pH threshold of both fusion and E1 homotrimer formation. Thus H3 plays a role in regulation of the low pH-dependent refolding of E1 during membrane fusion.

Inhibition of DIII fold-back and hairpin formation. A key step in formation of the final E1 HT is the packing of DIII and the stem region against the central trimer formed by domains I and II. The presence of exogenous DIII during the low pH-triggered fusion reaction specifically inhibits alphavirus and flavivirus membrane fusion and infection [65]. Studies with SFV show that fusion is blocked prior to the lipid mixing step, and that DIII inhibitory activity is increased by the presence of the stem region. Biochemical studies show that DIII acts by binding to the central trimer, thus preventing the fold-back of endogenous DIII and the formation of the final hairpin. DIII binding is specific, rapid, and stable, suggesting a high affinity interaction [65]. Although the alphavirus and flavivirus fusion reactions are very rapid, within this process the core trimer is a relatively accessible and long-lived intermediate, suggesting the possibility of targeting the DIII-core trimer binding step in anti-viral strategies.

Residues involved in E1 core trimer formation. Formation of the core trimer appears to be a critical step in E1 refolding and membrane fusion. The central trimer interface contains a highly conserved aspartate residue D188. Although there are extensive contacts between E1proteins in the homotrimer, a single point mutation of D188 to lysine inhibits E1 trimerization, membrane fusion, and virus infection [64]. Dimer dissociation and E1-membrane interaction are unimpaired, while formation of the core trimer that interacts with exogenous DIII is blocked. Thus the results with this mutant support a model based on initial membrane insertion of an E1 monomer and subsequent formation of a trimer core that binds DIII during hairpin formation and fusion.

### 5.4. Cholesterol dependence

Cholesterol-depleted mosquito cells were used to select for SFV mutants with decreased cholesterol-dependence for growth. Three such *srf* mutants (sterol requirement in function) have been isolated [86,156]. All three mutants have single amino acid changes in E1 that decrease the cholesterol-requirement: proline 226 to serine for *srf*-3, leucine 44 to phenylalanine for *srf*-4, and valine 178 to alanine in the case of *srf*-5. No mutations were found in the other structural proteins, thus confirming the role of E1 in SFV fusion and cholesterol dependence. *srf*-3 was independently isolated 8 times, and therefore identifies a preferred site that confers cholesterol independence. The *srf*-3 mutation P226S is located on the ij loop at the tip of domain II adjacent to the fusion loop, while the *srf*-4 and *srf*-5 mutations both lie in the hinge region of domain II more distant from the membrane-interacting region. The juxtaposition of the *srf*-4 and *srf*-5 mutations in the structure of E1 is in keeping with the finding that when both mutations are present in E1, folding and transport to the plasma membrane are impaired at 37 °C [156].

All three *srf* mutants show more efficient growth, fusion, and budding in the absence of cholesterol than the wt virus [86,156]. Fusion of the mutants with the plasma membrane of depleted cells is increased by 100 to 1000-fold, although all three mutants fuse and infect with maximum efficiency on cholesterol-containing cells, and thus are not fully sterol-independent. Liposome fusion studies reveal that the *srf*-4 and *srf*-5 mutants do not require sphingolipid for either hemi-fusion or complete fusion [156]. Interestingly, although both *srf*-4 and *srf*-5 form functional, trypsin-resistant E1 homotrimers at acid pH, these trimers are unstable in SDS. The acid-induced conformational changes in E1 are promoted by cholesterol liposomes for the wt virus and are less cholesterol-dependent for *srf*-3 [157]. Thus the P226S mutation appears to enhance cholesterol-independent fusion by increasing the cholesterol independence of E1 conformational changes in the fusion pathway.

SIN also requires cholesterol for efficient fusion and budding, and the cholesterol independence of SIN growth, infection, fusion and budding are increased when the sequence of the SIN ij loop (AKN) is changed to that of *srf*-3 (SGM) [87]. Point mutations show that the regulation of cholesterol independence is more complex than simply the presence of a serine at position 226, and that, depending on the adjacent sequence, A226 can also confer cholesterol independence. Thus, the overall conformation of the ij loop and the residue at 226 are both important factors [77,87].

During a recent extensive epidemic of infection by the alphavirus chikungunya virus, viruses with an E1 A226V mutation were preferentially isolated [158]. The A226V virus shows increased replication in and transmission by the mosquito vector *Aedes albopictus*, the predominant vector during the epidemic [159,160]. This mutation also makes the virus more cholesterol-dependent for growth [160]. It will be interesting to determine whether there is a connection between vector adaptation and cholesterol dependence, and the role of other mutations in modulating these effects [161].

### 5.5. Role of the E1 ij loop

The effects of the ij loop on alphavirus cholesterol dependence and the comparable protease susceptibility properties of the cd and ij loops in the E1 homotrimer suggested a potential role for the ij loop in alphavirus fusion. Sequence comparisons reveal that a histidine is found at position 230 (SFV numbering) in the ij loop of all alphavirus E1 sequences in the database, including those of the more distantly related fish alphaviruses [63]. Although the mutant virus particles resemble wild type in morphology, an SFV E1 H230A point mutant is non-infectious, and is blocked in both content and lipid mixing assays of membrane fusion [63]. The mutant virus binds and is endocytosed normally by cells. It responds to acid pH by dissociation of the E2/E1 dimer, exposure of the fusion loop, E1-target membrane insertion, exposure of acid-specific epitopes, and formation of a trypsin- and SDS-resistant homotrimer. Both the pH- and cholesterol-dependence of the E1 conformational changes are unaltered. Thus, the H230A mutation affects a late step in fusion preceding the merging of the outer leaflets of viral and target membranes.

A variety of second-site mutations in E1 can rescue the lethal H230A mutant [162]. Interestingly, all three of the *srf* mutations rescue H230A, although there is no correlation between the cholesterol requirements of the revertants and their ability to rescue H230A fusion and infection. The second-site resuscitating mutations are all located in DII, clustered within the E1 hinge region, in the ij and fusion loops at the membrane-interacting DII tip, and within the groove where the E1 stem would pack. The mutations suggest functional connections between these regions of E1 during refolding to the final homotrimer.

### 5.6. Roles of the E1 stem and transmembrane domains

The E1 stem domain. The E1* ectodomain contains approximately the N-terminal third of the stem region (Figure 1). This segment is disordered and thus not visualized in the pH 7 E1* structure [10,12], and becomes ordered and packs against the homotrimer core in the E1*HT structure [118]. An antibody to the N-terminal third of the stem demonstrates that this region becomes exposed upon dimer dissociation and that its packing against the trimer core is a relatively late step in formation of the final post-fusion hairpin, occurring after the fold-back of DIII [163]. The length of the stem in alphavirus E1 is strictly maintained and there are several highly conserved residues in this region. Mutagenesis studies of the SFV stem show that while the stem is important for E2-E1 dimerization and virus assembly, there are no specific stem sequence requirements for membrane fusion [164]. A minimal length is required, presumably to allow DIII fold-back and span the distance between DIII and the transmembrane domain in the post-fusion conformation of E1. The lack of a specific sequence requirement for the stem interaction during fusion suggests that stem peptides alone may not be potent alphavirus fusion inhibitors [c.f., reference 163], but clearly the presence of the stem stabilizes the HT and increases binding and inhibition by exogenous DIII [65].

The E1 transmembrane domain. Studies of the class I virus proteins have shown that the TM domain and the cytoplasmic tail of these fusion proteins can play important roles in fusion [reviewed in 165]. SFV E1 has a predicted TM domain of about 23 residues, and a predicted cytoplasmic tail that is relatively short [11]. A mutant with a deletion of the two arginine residues in the SFV E1 cytoplasmic tail fuses with a pH dependence similar to that of wt SFV, although its budding is somewhat impaired [166]. Thus, to date there is no evidence for a sequence-specific role of the alphavirus E1 cytoplasmic tail in fusion.

The alphavirus E1 and E2 TM domains associate in the heterodimer and in the virus particle [9,11]. Although the sequences of these two TM domains are not conserved among different alphaviruses, a number of studies have demonstrated that there are important and specific interactions between the E1and E2 TM domains that affect dimer stability and virus budding and assembly [142,167–169]. The E1 TM domain has two highly conserved glycines plus three other glycine residues that are not conserved. Mutation of the two conserved glycines decreases dimer stability, promotes E1 homotrimer formation, and decreases fusion activity [168]. Replacement of all 5 glycine residues did not affect virus growth but caused a modest decrease in fusion kinetics and efficiency *in vitro* [170]. Thus, while the sequence of the E1 TM domain is not critical for fusion, the correct interaction of the dimer helps to regulate E1 conformational changes and optimize fusion.

### 5.7. In vitro reconstitution of trimerization

Truncated versions of SFV E1 containing only domains I and II (DI/II) were expressed and used to reconstitute steps in the trimerization reaction *in vitro* [116]. E1 DI/II inserts stably into target membranes in a reaction dependent on low pH and membrane cholesterol. Electron microscopy shows that the membrane-inserted DI/II is trimeric and that, similar to E1*, these truncated trimers interact to form rings of 5–6 trimers and patches of hexagonal lattice. The DI/II trimers have membrane-deforming activity and generate elongated membrane tubules from spherical liposomes. These core trimers specifically bind exogenous DIII, thus reconstituting hairpin formation. Binding is stabilized by the presence of the stem region, and unlike the overall E1 refolding reaction, the DIII binding step is pH-independent. This reconstituted system thus suggests that the DI/II regions of E1 are responsible for the low pH-dependent steps in E1 refolding. The cooperative inter-trimer interactions and membrane bending activity observed in the absence of hairpin formation suggests the importance of fusion loop contacts during membrane fusion.

## 6. Future directions

Fundamental advances have been made in understanding the structural basis of the protein rearrangements during alphavirus fusion. Many key questions remain, but now they can be addressed in the context of the pre- and post-fusion structures of the alphavirus E1 protein. It will be important to address the role of the specific steps in the E1 conformational change in mediating individual steps in fusion. Is fusion essentially driven by the trimer refolding process or are there additional steps still to be defined? What is the role of the observed cooperative inter-trimer interactions? Is there a functional difference in E1 proteins at the five-fold axis of symmetry *vs.* other E1 proteins on the virus surface [171]? How does the overall virus structure reorganize during fusion to permit dissociation of the heterodimer and movement of the E1 and E2 proteins and their TM domains? Does E2 play further roles in membrane fusion in addition to the dimer regulation process described here? How is membrane insertion triggered and what is the role of specific lipids in this process? Do the E1 fusion loop and TM domain interact in the final fused membrane, and is this interaction critical for fusion? What are the similarities and differences between the alphavirus and flavivirus fusion mechanisms, and are there additional structurally-related fusion proteins in other virus groups? Lastly, can inhibitors of the alphavirus and related fusion reactions be identified based on inhibiting intra-trimer or inter-trimer interactions during fusion? The combination of structural analysis, biochemistry, and molecular virology will contribute to understanding these questions in the future, and may yield important inhibitors of fusion for both experimental and therapeutic use.

## Figures and Tables

**Table 1.** Evidence for alphavirus infection via endocytosis and low pH-triggered membrane fusion.

**Observation**	**Selected References**
Morphological and biochemical observation of endocytic uptake	[39,43]
Infection from within endosomes	[44]
Infection/fusion inhibited by dominant-negative inhibitors of endocytosis: dynamin eps15 rab-5	[45,46] [47] [48]
Infection/fusion inhibited by weak bases (e.g. NH_4_Cl, chloroquine)	[39,44,49]
Infection/fusion inhibited by ionophores (e.g. monensin)	[50,51]
Infection/fusion inhibited by vacuolar proton pump inhibitors (e.g. bafilomycin, concanamycin)	[46,49,52]
Specific low pH-dependence of pseudotype infection	[53]
Low pH-dependent cell-cell fusion	[54–57]
Low pH-dependent virus fusion with liposomes	[58–60]
Low pH-dependent fusion pore formation	[56,61]
Mutations block both membrane fusion *in vitro* and virus infection *in vivo*	[62–64]
Exogenous DIII blocks both fusion and infection	[65]

**Table 2.** Evidence for role of the alphavirus E1 homotrimer in fusion.

**Observation**	**Selected References**
Timing during virus uptake *in vivo*	[51,100]
*In vivo* formation requires endosomal acidification	[100]
Block in homotrimer formation in fusion-defective E1 mutants G91D, D188K	[62,64]
Timing during virus fusion *in vitro*	[58,101]
Correlation with pH-dependence of virus fusion	[35,74]
Exogenous DIII blocks E1 hairpin formation and fusion	[65]

**Table 3.** Summary of alphavirus mutations that compensate for altered E1-E2 interactions.

**Virus used for selection**	**Glycoproteins**			**References**
	**E3**	**E2**	**E1**	
wt/p62 (SFV)		N7D, N77D, A121E, H170Y, L221Q, R244G, R244K, R250G	V11A, T159A	[140,141]
SHQL(SFV)	H64R	Q4R, R244I, R244K		[139]
TRSB-N(SIN)	C25R	D82G, H169L, P191T, T198M, E216gG, N239H		[35,137]
VEE deletion		L243N	F253S	[135]
SIN E2/RRV 6k+E1(chimera)		D72N, S118N, K131E, I150L, V237F, L243S, D248Y, I380S	S310F, F399S, Q411L, I423L, C433R	[133,142,143]
